# Predictors of switching from beta-blockers to other anti-hypertensive drugs: a review of records of 19,177 Chinese patients seen in public primary care clinics in the New Territory East, Hong Kong

**DOI:** 10.1186/1447-056X-10-10

**Published:** 2011-07-27

**Authors:** Martin CS Wong, Harry HX Wang, Johnny Y Jiang, Stephen Leeder, Sian M Griffiths

**Affiliations:** 1 School of Public Health and Primary Care, Faculty of Medicine, Chinese University of Hong Kong; HKSAR, China; 2 Chinese Academy of Medical Science & Peking Union Medical College, Beijing, China; 3 Menzies Centre for Health Policy, School of Public Health, The University of Sydney, Australia

**Keywords:** beta-blocker, switching, pharmacoepidemiology, family practice

## Abstract

**Background:**

Beta-blocker drugs are commonly used in family practice and studies showed that they were the most popularly prescribed medications among all antihypertensive agents. This study aimed to identify the factors associated with medication switching from a beta-blocker to another antihypertensive drug among Chinese patients.

**Methods:**

We used a validated database which consisted of the demographic and clinical information of all Chinese patients prescribed a beta-blocker from any public, family practice clinics between 01 Jan 2004 to 30 June 2007 in one large Territory of Hong Kong. The proportion of patients switched from beta-blockers to another antihypertensive agent 180 days within their first prescription was studied, and the factors associated with medication switching were evaluated by using multivariate regression analyses.

**Results:**

From 19,177 eligible subjects with a mean age of 59.1 years, 763 (4.0%) were switched from their beta-blockers within 180 days of commencing therapy. A binary logistic regression model used medication switching as the outcome variable and controlled for age, gender, socioeconomic status, clinic setting (general out-patient clinics, family medicine specialist clinic or staff clinics), district of residence, visit type (new vs. follow-up attendance), the number of concomitant co-morbidities, and the calendar year of prescription. It was found that older patients (age 50-59 years: adjusted odds ratio [AOR] 1.38, 95% C.I. 1.12-1.70; p = 0.002; age 60-69 years: AOR 1.63 95% C.I. 1.30-2.04, p < 0.001; age ≥ 70 years: AOR 1.82, 95% C.I. 1.46-2.26, p < 0.001; referent age < 50 years) and new visitors (AOR 0.57, 95% C.I. 0.48-0.68, p < 0.001) were more likely to have their medication switched.

**Conclusions:**

Closer monitoring of the medication taking behavior among the older patients and the new clinic visitors prescribed a beta-blocker is warranted. Future studies should evaluate the reasons of drug switching.

## Background

Hypertension is a global health problem and represents one of the most important, modifiable risk factors for renal and cardiovascular diseases [1] It affects more than 30% of the general US population [2], and estimates of the cost of the disorder are around $66.4 billion worldwide in 2007 [3]. Its prevalence is high over the globe, including Asia Pacific countries [4]. Despite the effectiveness of antihypertensive pharmacotherapy to mitigate its complications [5,6], the control of hypertension remained suboptimal [7]. In family practice, its total yearly costs were approximately £76.5 million, of which £26.9 million was attributed to patients who discontinue or switch their pharmacotherapy [8].

Beta-blockers are commonly used in family practice to manage a wide variety of chronic diseases, including hypertension. Together with calcium channel blockers, beta-blockers are one of the most commonly prescribed antihypertensive agents in primary care settings [9]. The clinical guidelines used in the clinics of the public sector in Hong Kong were mainly from the 7^th ^Joint National Committee Seventh Report. Therefore, the major antihypertensive drug classes were regarded as equally acceptable as first-line prescriptions for management of arterial hypertension since 2003 [10]. In our recent studies on switching of antihypertensive drug therapies in Hong Kong, we found that among more than 93,000 subjects with hypertension, 5.7% switched their medications to another antihypertensive drug class within 180 days of their prescriptions [11]. Subjects at higher risks of medication switching were evaluated, and the risk factors included young age, female gender, attendance in family medicine clinics, new visitors and those prescribed thiazide diuretics as well [11]. However when we conducted stratified analysis of each antihypertensive drug class in the same group of patients, the associated factors of medication switching were different which signified a drug-class specific heterogeneity [12,13]. Beta-blocker is a distinct group of antihypertensive agent which has different efficacy depending on demographic groups and other comorbid conditions [14].

Previous studies among Caucasian populations have identified the risk factors associated with antihypertensive drug switching [15-19]. Nevertheless, ethnicity might be a factor which limits the generalizability of these findings, since patients in the Asia Pacific region might have a different profile of medication switching due to different pharmacological actions of beta-blockers [20,21]. In particular, studies on patients of Chinese ethnicity are particularly scarce despite the fact that they constitute one-fifth of the world's population. Chinese patients were shown to be more sensitive to beta-blockade effect when compared to white patients [22].

The primary objective of this study is to identify the independent factors associated with switching of beta-blockers in family practice. We tested the association between age, gender, socioeconomic status, service setting (general out-patient clinic [GOPC] or family medicine specialist clinic [FMSC] or staff clinic), district of residence, visit type (initial or follow-up visits) and the number of co-morbidities, which were also the variables tested in our previous studies [11-13].

## Methods

### Data Source

A detailed methodology has been presented elsewhere [9,11,23]. The Clinical Data Analysis and Reporting System (CDARS) of the Hospital Authority (HA) of Hong Kong consists of 7 million patient records, 1 million annual admissions and 13 million ambulatory visits, with research as one of its objectives [24]. The computerized system consists of patients' demographic and clinical information, medical diagnoses in the forms of International Classification of Primary Care version 2 (ICPC-2) codes, types of clinical services (location) and details on medication prescriptions. This database has previously been investigated and the demographic (100%) and prescription details were 99.98%) complete [9].

When patients visit public primary care clinics in Hong Kong, they register with their identity documents. Socio-demographic information is recorded by the reception staff, and entered into the computer system as are drug prescription (with paper script back-up during computer down-times) for both public and private systems.. All drug items were checked by two qualified dispensers or pharmacists. Records are unified so that they are available at any primary care clinics where the patient presents. Clinical guidelines assist physicians to enter the clinical diagnoses for each consultation using ICPC-2 codes.

This study retrieved patient information from the New Territory East cluster (NTE) of Hong Kong, which offers primary healthcare services to approximately 1.3 million residents, or 17.2% of the Hong Kong population [25]. The territory is further divided into three geographical regions, namely Shatin (urbanized), Taipo and North districts (rural). The median monthly household incomes of residents in these three regions in 2006 were US$2,510, US$2,338 and US$2,078, respectively, which were comparable to the Hong Kong-wide figure of US$2,240 [25]. The median ages in these three regions were similar (38-39 years: median age of 39 years for Hong Kong). We obtained ethical approval from the Survey and Behavioural Research Ethics Committee of the Chinese University of Hong Kong. All patients were anonymized and their identities were replaced by unique identifiers, hence informed consent was not required.

### Definition of the cohort

All adult patients aged ≥ 18 years who attended a primary care clinic in the NTE region during the study period January, 2004 to June, 2007, for whom at least one antihypertensive medication was prescribed, were included in our analysis. Each patient was assigned the number of co-morbidities according to the number of concomitant cardiovascular disorders other than ICPC-2 K86 [uncomplicated hypertension] (e.g., diabetes mellitus, cerebrovascular diseases, lipid disorders) and medical conditions which could potentially influence prescription of a particular class of antihypertensive drug (e.g., gout, asthma, ischemic heart diseases, benign prostatic hypertrophy). Table 1 shows a complete list of these medical conditions with their respective ICPC-2 codes.

**Table 1:** Comorbidities according to the respective International Classification of Primary Care (ICPC) Codes

ICPC codes	Disease entity
T90	Diabetes Mellitus
T901	Impaired glucose tolerance
T92	Gout
T93	Lipid disorders
K90	Stroke/cerebrovascular accident
K91	Cerebrovascular disease
K74	Ischemic Heart Disease with angina
K76	Ischemic Heart Disease without angina
K75	Acute Myocardial Infarction
K77	Heart Failure
K84	Heart Disease, other
K99	Cardiovascular disease, other
R79	Chronic Bronchitis
R95	Chronic Obstructive Pulmonary Disease
R96	Asthma
U14	Kidney symptoms/complaints
U88	Glomerulonephritis/nephrosis
Y85	Benign Prostatic Hypertrophy
U78	Benign Neoplasm Urinary Tract
Y79	Benign/unspecified neoplasm, male genital

### Exposure to drugs and assessment of switching

The rate of medication switching within 180 days of their prescription was computed. We defined drug switching as "the absence of a refill prescription in all subsequent clinic visits with issuance of another antihypertensive drug of a different antihypertensive drug class within the time frame of 180 days since the first prescription date" [11]. A time frame of 180 days was used instead of 90 days since the durations of antihypertensive prescriptions were variable, ranging from 14 to 168 days (median 56 days).

### Covariates and statistical analysis

We used the same variables in a previous study [11] to test for associations with the primary outcome, namely medication switching 180 days within their prescriptions. The covariates included age, gender, socioeconomic status in terms of fee-payers vs. fee-waivers, service type, district of residence, visit type, the number of comorbidities and the calendar years of prescriptions. Fee waivers are mainly subjects receiving social security allowances from the government. To apply for fee waiving, applicants will receive a comprehensive assessment by a professional in the social welfare sector in order to certify their inability to pay for clinical consultation fees. Each consultation charges US$5.77, including investigation and medication fees. We defined new visitors as patients who have never been prescribed any antihypertensive medications in any clinics under HA. One unconditional, binary logistic regression analysis was conducted of all eligible patients, and all the predictor variables were entered into the regression models. The Statistical Package for Social Sciences version 15.0 (SPSS Incorporation, Chicago Illinois) was used for all data analysis. All p values ≤ 0.05 were regarded as statistically significant.

## Results

A total of 19,177 eligible patients were included in the present study. 41.5% were aged ≥ 60 years, and the majority were female (62.8%) (Table 2). Most were fee-payers (73.5%), attended GOPCs for the consultations (90.2%), lived in more urbanized regions (Shatin and Taipo: 70.8%), and were first-ever visitors (61.6%). Among them, 83.2% had no co-morbidities, and most prescriptions were issued in the calendar years 2004 (51.2%) and 2005 (22.7%).

**Table 2:** Patient characteristics (N = 19,177)

	**No**.	%
**Age (years)**		
< 50	5,327	27.8
50-59	5,885	30.7
60-69	3,863	20.1
≥ 70	4,102	21.4
		
**Gender**		
Male	7,133	37.2
Female	12,044	62.8
		
**Payment status**		
Fee waivers	5,088	26.5
Fee payers	14,089	73.5
		
**Service type**		
GOPC	17,294	90.2
FMSC	1,704	8.9
Staff clinic	179	0.9
		
**District of residence**		
Shatin	8,905	46.4
Taipo	4,684	24.4
North	4,246	22.1
Others	1,342	7.0
		
**Visit type**		
New	11,817	61.6
Old	7,360	38.4
		
**Comorbidities**		
0	15,952	83.2
1	2,774	14.5
2	432	2.3
> 3	19	0.1
		
**Calendar years of prescription**		
2004	9,826	51.2
2005	4,360	22.7
2006	3,402	17.7
2007	1,589	8.3

GOPC: General Out-patient Clinic; FMSC: Family Medicine Specialist Clinic; CCB: Calcium Channel Blockers; All the percentages are across columns

Table 3 shows the characteristics of patients who switched compared with those who continued their beta-blocker prescriptions. A total of 4.0% switched their medication within 180 days. When compared with drug continuers, those who switched their prescriptions were older, more were female (64.0% vs. 62.8%), fee payers (73.9% vs. 73.4%), and visitors of GOPCs (91.9% vs. 90.1%) (all p < 0.001). In addition, more patients who switched lived in more rural districts (the North region; 24.4% vs. 22.0%), were new visitors (74.7% vs. 61.1%) and had more co-morbidities (17.3% vs. 16.7%) (all p < 0.001).

**Table 3:** Characteristics of beta-blockers switchers and non-switchers (N = 19,177)

	Switchers (n = 763)		non-switchers (n = 18,414)		p
	**No**.	%	**No**.	%	
**Age (years)**					
< 50	158	20.7	5,169	28.1	< 0.001
50-59	229	30.0	5,656	30.7	
60-69	171	22.4	3,692	20.0	
≥ 70	205	26.9	3,897	21.2	
					
**Gender**					
Male	275	36.0	6,858	37.2	< 0.001
Female	488	64.0	11,556	62.8	
					
**Payment status**					
Fee waivers	199	26.1	4,889	26.6	< 0.001
Fee payers	564	73.9	13,525	73.4	
					
**Service type**					
GOPC	701	91.9	16,593	90.1	< 0.001
FMSC	59	7.7	1,645	8.9	
Staff clinic	3	0.4	176	1.0	
					
**District of residence**					
Shatin	357	46.8	8,548	46.4	< 0.001
Taipo	177	23.2	4,507	24.5	
North	186	24.4	4,060	22.0	
Others	43	5.6	1,299	7.1	
					
**Visit type**					
New	570	74.7	11,247	61.1	< 0.001
Old	193	25.3	7,167	38.9	
					
**Comorbidities**					
0	630	82.6	15,322	83.2	< 0.001
1	114	14.9	2,660	14.4	
≥ 2	19	2.5	432	2.3	
					
**Calendar years of prescription**					
2004	279	36.6	9,547	51.8	< 0.001
2005	227	29.8	4,133	22.4	
2006	189	24.8	3,213	17.4	
2007	68	8.9	1,521	8.3	

GOPC: General Out-patient Clinic; FMSC: Family Medicine Specialist Clinic. All the percentages were across columns.

From a binary logistic regression model, patients who were older (≥ 50 years; adjusted odds ratio [AOR] ranged from 1.38 to 1.82) and new clinic visitors (AOR for follow-up visitors = 0.57, 95% C.I. 0.48 to 0.68, p < 0.001) were significantly more likely to have their medication switched (Table 4). There were no statistically significant associations detected between other covariates with the outcome variable. When we used 240 days and 360 days as the yardsticks of medication switching and re-conducted the regression analyses, similar significant associations were detected for age and visit type. There existed no multi-colinearity for the regression models, suggesting their statistical robustness.

**Table 4:** Factors associated with switching of beta-blockers (N = 19,177, r^2 ^= 0

	**No**.	%	Adjusted odds ratios (95% C.I.)	p
**Age**				
< 50	158	3.0	1.00 (reference)	
50-59	229	3.9	**1.38 (1.12-1.70)**	**0.002**
60-69	171	4.4	**1.63 (1.30-2.04)**	**< 0.001**
≥ 70	205	5.0	**1.82 (1.46-2.26)**	**< 0.001**
				
**Gender**				
Female	275	2.3	1.00 (reference)	
Male	488	6.8	0.89 (0.76-1.03)	0.125
				
**Payment status**				
Fee waivers	199	3.9	1.00 (reference)	
Fee payers	564	4.0	1.02 (0.87-1.21)	0.792
				
**Service type**				
GOPC	701	4.1	1.00 (reference)	
FMSC	59	3.5	0.97 (0.73-1.28)	0.817
Staff clinic	3	1.7	0.47 (0.15-1.48)	0.196
				
**District of residence**				
Shatin	357	4.0	1.00 (reference)	
Taipo	177	3.8	0.95 (0.79-1.15)	0.606
North	186	4.4	1.07 (0.88-1.29)	0.505
Others	43	3.2	0.82 (0.59-1.13)	0.223
				
**Visit type**				
New	570	4.8	1.00 (reference)	
Old	193	2.6	**0.57 (0.48-0.68)**	**< 0.001**
				
**Comorbidities**				
0	630	3.9	1.00 (reference)	
1	114	4.1	1.17 (0.95-1.45)	0.150
≥ 2	19	4.2	1.23 (0.76-2.00)	0.396

GOPC: General Out-patient Clinic; FMSC: Family Medicine Specialist Clinic; CCB: Calcium Channel Blockers; RAS: Drugs acting on the rennin-angiotensin system. %: represents the percentages of patients with drugs discontinued.

## Discussion

From this retrospective cohort consisting of 19,177 Chinese patients, we reported that older age and first clinic visits were significant predictors of medication switching. The other factors previously shown to be associated with antihypertensive drug switching [11], including gender, service type, district of residence, visit type and co-morbidities, were no longer statistical significant. Thus far there were no studies of this scale which evaluated the factors associated with switching from a beta-blocker to another antihypertensive prescription.

The mechanism where beta-blockers lower blood pressure could be multifactorial [26]. Some suggested mechanisms included diminution of sympathetic outflow mediated by the effect of the central nervous system, and reduced cardiac output accompanied by increased peripheral resistance [27]. Among the elderly, hypertension is characterized by compromised cardiac output and escalated peripheral vascular resistance [28,29]. Beta-blockers used in the long term may lead to homeostatic adjustments that can improve cardiac output but will then be depressed when compared with the original state. Furthermore, there has been evidence that lipophilic beta-blockers can easily cross the blood-brain barrier, which induces more depression, sedation and sexual dysfunction in older subjects [30]. In addition, beta-blockers have strong potentials to induce conduction abnormalities, undesirable bradycardia, and development of heart failure especially among elderly with left ventricular dysfunction [30-32].

Evidence points towards the relative ineffectiveness of beta-blockers as a treatment regimen for hypertension among the elderly populations. For instance, one meta-analysis including more than 16,000 elderly subjects 60 years or older reported that beta-blockers were incapable to prevent coronary heart diseases, fatal or non-fatal myocardial infarctions [33]. Also, when compared with other antihypertensive therapies, beta-blockers were the least effective in reducing left ventricular mass in the elderly although the effects on blood pressure lowering were similar across major antihypertensive drug classes [34-36]. Therefore with an exception of the Joint National Committee 7^th ^report, beta-blocker has not been recommended as a first-line agent in the elderly without any concomitant disorders like congestive heart failure or myocardial infarction [37].

Medication switching is essentially a physician-initiated action, but the patient could have influence. The physician might perceive the initial beta-blocker regimen is better replaced by a more efficacious antihypertensive medication to control blood pressure or other cardiovascular risk factors. For instance, elderly patients are more likely to suffer from poorly controlled diabetes and impaired glucose tolerance, and switching of beta-blockers to another agent is warranted. It can also be patient-driven, like a complaint of intolerable adverse events. Our findings that older subjects were more likely to have their beta-blockers switched could be explained by either or both of the above reasons. That the new visitors were more likely to have their medication switched is not surprising, as the follow-up patients might already have refilled their medications for at least once and should be arguably free of adverse effects when compared with the new clinic attendees.

Our study included a large number of Chinese subjects. The good dispensing practice in the primary care out-patient clinics allowed an accurate analysis of prescription patterns free of recall biases. However some limitations should be mentioned. Firstly this study included a Chinese population from only one geographical region out of seven in Hong Kong, although the demographic features of our samples are similar to all residents in Hong Kong. In addition, we captured only short-term switching profiles, and longer term prescription changes could not be assessed. Furthermore, the reasons of medication switching could not be evaluated from retrospective database analyses, and there remained some confounding factors like blood pressure level, body mass index, lifestyle habits and attitudes towards taking of chronic medications which could not be controlled for.

## Conclusions

The implication of this study is that more careful practice of prescribing beta-blockers is needed particularly among the elderly and also the new visitors, who are more likely to be drug-naive. Translated into clinical practice, family physicians should attempt closer monitoring of the medication taking behavior of their patients with these risk factors. In the broader context of primary care, a multidisciplinary team of healthcare professionals should collaborate and strengthen medication management in the community. This could involve community pharmacists who can give timely advice to patients regarding medication counseling and support.

The future direction of research should be targeted towards the reasons of medication switching, and to what degree family physicians were influenced by evidence-based guidelines regarding prescription of first-line antihypertensive agents.

## List of abbreviations

AOR: adjusted odds ratios; CDARS: Clinical Data Analysis and Reporting System; FMSC: Family Medicine Specialist Clinic; GOPC: General Out-Patient Clinic; HA: Hospital Authority; ICPC-2: International Classification of Primary Care, version 2; NTE: New Territory East

## Competing interests

The authors declare that they have no competing interests.

## Authors' contributions

MCSW participated in the study conception and design, acquisition of data, coordinated the study, and drafted the manuscript. HHXW and JJY conducted literature search, performed statistical analysis and participated in data interpretation. SL and SG critically revised the manuscript and contributed to intellectual advice. All authors read and approved the final manuscript.

## Authors' information

not applicable
